# *Gentiana daofuensis* (Gentianaceae), a new species from Sichuan, China

**DOI:** 10.3897/phytokeys.272.183828

**Published:** 2026-03-18

**Authors:** Shan-Shan Sun, Yuan-Meng Guo, Xin-Yue Zhang, Wen-Jie Yi, Shu-Han Xu, Peng-Cheng Fu

**Affiliations:** 1 School of Life Science, Luoyang Normal University, Luoyang, China School of Life Science, Luoyang Normal University Luoyang China

**Keywords:** Chloroplast genome, *
Gentiana
*, Qinghai-Tibet Plateau, section *Chondrophyllae*

## Abstract

*Gentiana daofuensis*, a new species of the family Gentianaceae, is described and illustrated here. Currently, this species is known only from Daofu County, Sichuan Province, China. Phylogenetic analyses based on chloroplast genome data indicate that it belongs to *G.* section *Chondrophyllae* s.l. Morphologically and genetically, it is closely related to *G. crassuloides* but can be clearly distinguished from the latter by its shorter calyx and pale blue-purple corolla with numerous dark blue short stripes or spots.

## Introduction

*Gentiana* (Gentianaceae) is a sub-cosmopolitan genus encompassing more than 360 species (Ho and Liu 2001). Due to considerable morphological variation, the genus is divided into 14 sections (Sun et al. 2026). Its main center of diversity lies in the Qinghai-Tibet Plateau (QTP) region, which has served as the primary source for subsequent dispersal to many mountainous regions worldwide (Ho and Liu 2001; Favre et al. 2016). Although widely distributed, only one species-rich section—section *Chondrophyllae* Bunge *sensu lato* (s.l.) (ca. 182 species, representing 51.7% of the genus; Favre et al. 2020)—is nearly globally distributed, while the remaining 13 sections are restricted to one or two continents (Ho and Liu 2001; Favre et al. 2016). Numerous gentians are cultivated in Europe and Japan for ornamental purposes (Rybczyński et al. 2015; Nishihara et al. 2018) and in China and Europe for medicinal applications (Zhou et al. 2010; Pasdaran et al. 2023; Yuan 2024).

Over the past two decades, eight new *Gentiana* species have been described from the QTP region (e.g., Ho and Liu 2010; Yang et al. 2020; Fu et al. 2021; Favre et al. 2022; Chen et al. 2024), and cryptic diversity has also been detected (Fu et al. 2024), indicating that the species diversity within *Gentiana* remains underestimated in this area.

During field surveys in 2023 and 2025 in Daofu County, Sichuan Province, located within the QTP region, we encountered an unknown *Gentiana* species. Phylogenetic analysis based on the chloroplast genome was conducted to infer its systematic position, followed by detailed morphological comparison with similar species. These results confirm that the plant represents a distinct new species belonging to section *Chondrophyllae* ser. *Orbiculatae* Marquand.

## Materials and methods

### Morphological analyses

The morphology of the new species was assessed through field observations of living plants and examination of specimens deposited at HNWP and the herbarium of Luoyang Normal University. Digital images of type specimens belonging to *Gentiana* section *Chondrophyllae* s.l. were consulted via JSTOR Global Plants (http://plants.jstor.org/) and the Chinese Virtual Herbarium (http://www.cvh.ac.cn/). Additional comparisons were made using collections held at HNWP and the herbarium of Luoyang Normal University. Morphological studies were carried out on dried specimens using stereomicroscopes, with measurements of various organs taken using rulers and metric vernier calipers. For each population of a given species, five to seven individuals were studied, and approximately 10 measurements were taken per trait. The results were visualized with function geom_boxplot in the R package ggplot2 (Wickham 2011).

### Phylogenetic study

Whole chloroplast genomes were obtained via genome-skimming sequencing. Total DNA was extracted from fresh silica-gel-dried leaves using a Dzup plant genomic DNA extraction kit (Sangon, Shanghai, China). Libraries were constructed for each material, multiplexed, and sequenced on the BGI DNBSEQ-T7 platform, yielding approximately 5 Gb of 150-bp paired-end reads per sample. Adapters and low-quality sequences were removed with Trimmomatic v.0.32 (Bolger et al. 2014), and read quality was assessed with FastQC v.0.11.9 (Andrews 2018). Chloroplast genomes were assembled using GetOrganelle v.1.7.7.1 (Jin et al. 2020) using the default parameters and annotated via PlastidHub (Zhang et al. 2025). Alongside newly generated data in this study, publicly available *Gentiana* chloroplast genomes were retrieved from Fu et al. (2022) and Sun et al. (2024). After excluding the second inverted repeat (IR) region, plastome sequences were aligned with MAFFT v.7.525 (Katoh et al. 2002). Phylogenetic relationships were inferred using maximum likelihood (ML) analysis. The best-fitting model of sequence evolution was selected with ModelFinder (Kalyaanamoorthy et al. 2017) under the AIC criterion. ML trees were constructed in IQ-TREE (Nguyen et al. 2015), with branch support evaluated using 1000 bootstrap replicates.

## Results and taxonomic treatment

### Gentiana daofuensis

Taxon classificationPlantaeGentianalesGentianaceae

Y.M.Yuan & P.C.Fu sp. nov.

A24D894C-2563-551A-81A2-E074569AC523

urn:lsid:ipni.org:names:77377804-1

Fig. 1

#### Type.

China • Sichuan Province: Daofu County, near Geka Village, alt. 3810 m, 101.31°N, 30.78°E, 23 Jul 2025, flowering and with few fruits, *P.C. Fu & S.S. Sun Fu2025075* (**holotype**: HNWP392646!; **isotype**: PE!).

#### Diagnosis.

*Gentiana daofuensis* is distinguishable from all other similar species of the genus by the combination of the reniform leaves on the upper stem and the pale blue-purple corolla with numerous dark blue short stripes or spots.

#### Etymology.

The specific epithet “*daofuensis*” is derived from the type locality of the new species, Daofu County, and the Latin suffix -ensis, indicating the place of origin or growth.

#### Vernacular name.

Chinese mandarin: dao fu xiao long dan (道孚小龙胆).

#### Description.

Annual herb, 3–16 cm tall. Stems ascending, often purplish-red, densely papillate, much branched from base, further repeatedly dichotomously branched. Basal leaves rapidly withering, blade ovate-elliptic or ovate, 5–12 × 3–6 mm, base rounded or cordate, apex acute and cuspidate with a recurved tip, margin thickly cartilaginous, midvein white cartilaginous, smooth, prominent beneath, petiole densely papillate on abaxial surface, 0.5–1 mm long. Stem leaves spreading or nearly perpendicular to the stem, widely spaced, petiole 0.5–1 mm, papillate, base rounded, margin thick cartilaginous, midvein prominent; middle and lower leaves ovate-triangular, 1.5–2 × 2.5–3 mm, apex acute and cuspidate with a recurved tip; upper leaves reniform, wider than long, 2–4 × 3–6 mm, apex rounded to truncate with a recurved tip. Flowers several, solitary at apex of branchlets; pedicel often purplish-red, densely papillate, 0.5–7 mm long; calyx tubular, green, 5–7 mm long, tube membranous, lobes recurved, uniform, reniform or broadly circular, 1.1–1.2 × 1.2–1.5 mm, base rounded to cordate, abruptly contracted, margin thick cartilaginous, apex rounded to truncate and mucronate with a recurved tip, midvein white, thickly cartilaginous and prominent. Corolla salverform, 1–1.4 cm long, 0.9–1.2 cm in diameter, 2.5–3 mm in diam. at throat; corolla tube narrowly tubular, base yellow to yellow-green, upper outside dark blue, inside pale blue-purple, with numerous dark blue short stripes or spots; lobes ovate, 1.5–2.3 mm long, margin entire, apex obtuse without a tip; plicae broadly ovate, 1–1.2 mm long, apex obtuse, margin entire or erose. Stamens inserted at middle of corolla tube, equal; filaments ca. 3 mm long, white; anthers narrowly oblong, ca. 0.8 mm long, yellow to violet; ovary oblong or elliptic, 3–4 mm long. Style linear, 1.5–2 mm long, white to pale green; stigma 2-lobed, reflexed, broadly linear, white to pale green. Capsule exserted or included, oblong or obovate-oblong, 6–9 mm; gynophore to 1 cm, stout. Seeds pale brown, oblong or elliptic, 1–1.2 mm in diameter, surface with dense fine reticulations.

**Figure 1.**
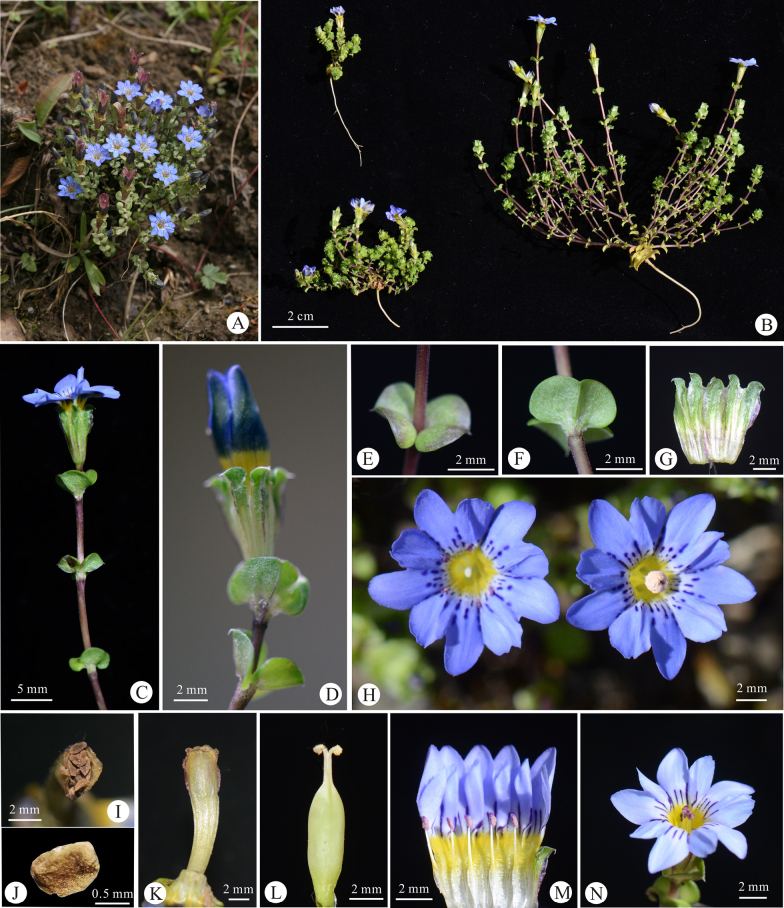
Morphological characteristics of *Gentiana daofuensis* sp. nov. **A**. Habit; **B**. Whole plants; **C**. Stem with flower at anthesis; **D**. Stem with mature flower bud; **E**. Leaf pair (front view); **F**. Leaf pair (reverse view); **G**. Calyx; **H**. Flower (front view); **I**. Fruit (front view); **J**. Seed; **K**. Fruit (side view); **L**. Pistil; **M**. Dissected flower; **N**. Flower. Photographs by Peng-Cheng Fu (**B–N**) and Yong-Ming Yuan (**A**).

#### Phenology.

*Gentiana daofuensis* has a long flowering and fruiting period. We have observed flowering and fruiting from July to September.

#### Distribution and habitat.

*Gentiana daofuensis* is currently only found in Daofu County, which is located at the southeastern edge of the Qinghai–Tibet Plateau. This species usually occurs at elevations between 3,600 and 3,900 meters and mainly grows in dry or rocky roadsides, hillsides, meadows, and scrub margins. It can also occasionally be found at forest margins.

#### Morphological analysis.

**Figure 2.**
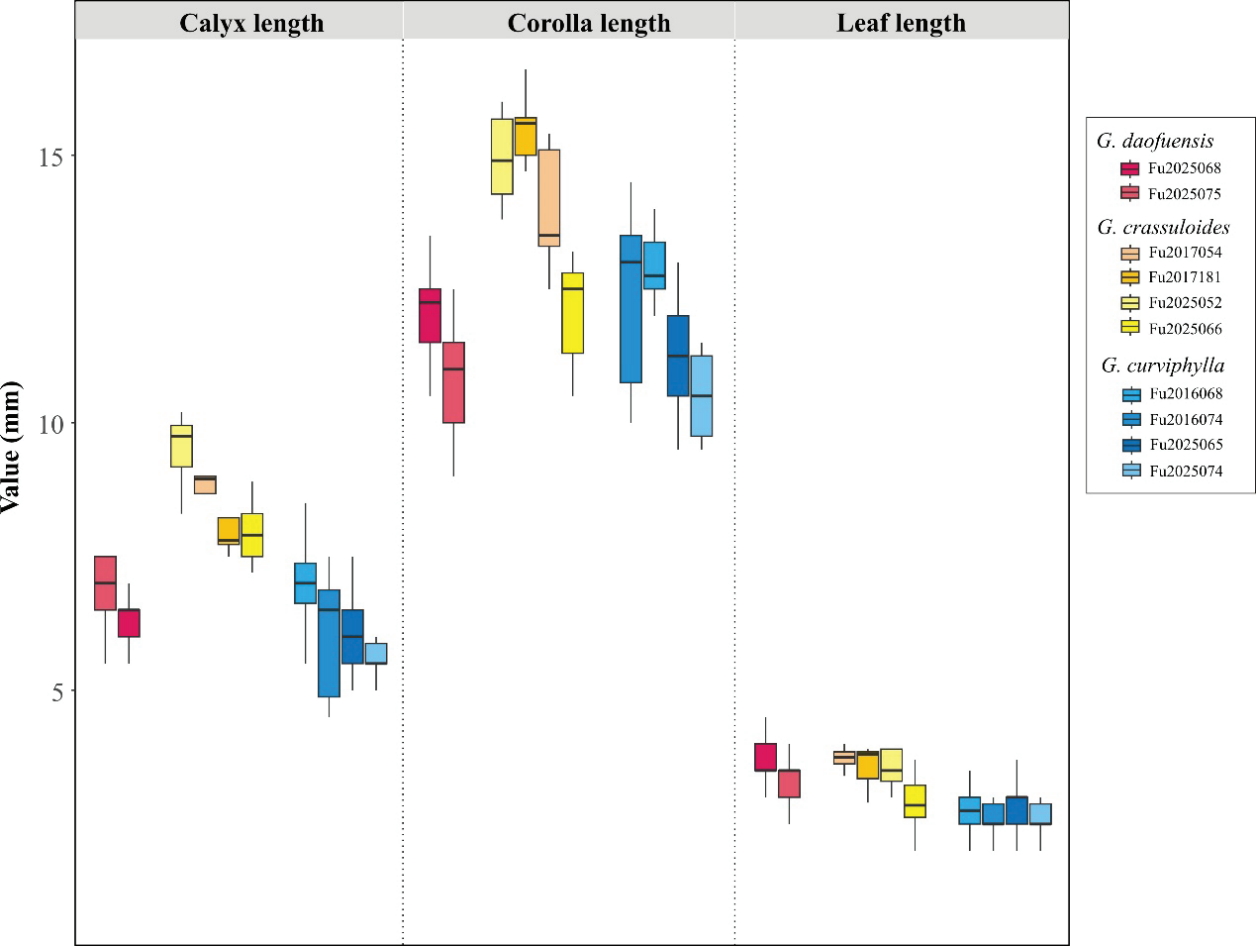
Boxplots of three key morphological characters (calyx length, corolla length, and leaf length) in *Gentiana daofuensis* sp. nov. and its two close relatives based on population surveys. Material studied: *Gentiana crassuloides*. China. Qinghai: Yushu County, alt. 4010 m, 97.20°N, 32.77°E, 12 Aug 2017, Fu2017054. Sichuan: Maerkang County, alt. 3340 m, 102.63°N, 31.88°E, 21 Aug 2017, Fu2017181; Maoxian County, alt. 3020 m, 103.74°N, 31.51°E, 21 Jul 2025, Fu2025052; Daofu County, alt. 3820 m, 101.58°N, 30.53°E, 23 Jul 2025, Fu2025066. *Gentiana curviphylla*. China. Sichuan: Ganzi County, alt. 3960 m, 101.18°N, 31.61°E, 12 Aug 2016, Fu2016068; Daofu County, alt. 3550 m, 101.13°N, 30.98°E, 13 Aug 2016, Fu2016074; Daofu County, alt. 3820 m, 101.58°N, 30.53°E, 23 Jul 2025, Fu2025065; Daofu County, alt. 3810 m, 101.31°N, 30.78°E, 23 Jul 2025, Fu2025074. All specimens are kept in the herbarium of Luoyang Normal University.

Morphologically, *G. daofuensis* is most similar to *G. crassuloides* Bureau & Franch. as well as *G. curviphylla* T.N.Ho. In total, we examined two populations of *G. daofuensis*, four of *G. crassuloides*, and four of *G. curviphylla*. Analysis of specimens from distinct localities for each species revealed clear morphological differences among the three species (Table 1; Fig. 2). Overall, *G. daofuensis* most closely resembles *G. crassuloides* within the genus but differs from the latter in its drier habitat preference, shorter calyx, paler blue color, and the presence of short stripes or spots on the corolla (Table 1; Fig. 2). *Gentiana daofuensis* generally has a shorter corolla than *G. crassuloides*, although overlap in length is sometimes observed (Fig. 2). *G. daofuensis* differs from *G. curviphylla* in the shape of the upper stem leaves and the paler color of the corolla lobes and in the narrower corolla tube at the throat (Table 1).

**Table 1.** Comparison of key characters among *Gentiana daofuensis*, *G. crassuloides*, and *G. curviphylla*. The information for the latter two species was retrieved from Flora of China (Ho and Pringle 1995).

Characters	*G. daofuensis*	*G. crassuloides*	*G. curviphylla*
Height	3–16 cm	3–6 cm	3–7 cm
Stem	often purplish-red	green	often purplish-red
Upper stem leaves	reniform, 2–4 × 3–6 mm, spreading	reniform, 1.5–4 × 2–4.5 mm, spreading	spatulate, 3–4 × 1.5–2 mm, recurved
Calyx length	5–7 mm	5–12 mm	4.5–6 mm
Corolla color	upper tube and lobes inside pale blue-purple, with dark blue short stripes or spots; tube base yellow to yellow-green	upper tube and lobes inside blue to blue-purple, without stripes or spots; tube base pale yellow-green	upper tube and lobes inside blue to blue-purple, with dark blue short stripes or spots; tube base pale yellow-green
Corolla length	10–14 mm	9–21 mm	10–12 mm
Corolla diam. at throat	2.5–3 mm	1.5–5 mm	4–6 mm
Corolla lobe length	1.5–2.3 mm	1.5–2.5 mm	2–2.5 mm
Habitat	dry or rocky roadside, hillside, meadow, and shrub margin	stream and river banks, grassland slopes, bogs, scrub, forests	grassland slopes, clearings in forests
Altitude	3,600–3,900 m	2,700–4,500 m	2,800–4,300 m
Fl. and Fr.	July to September	June to September	June to August
Distribution	W Sichuan	China (S & SE Xizang, NW Yunnan, W Sichuan, SE Qinghai, S Gansu, W Hubei), Bhutan, NW India, Nepal, Sikkim	W Sichuan, Yunnan

#### Molecular phylogenetics.

**Figure 3.**
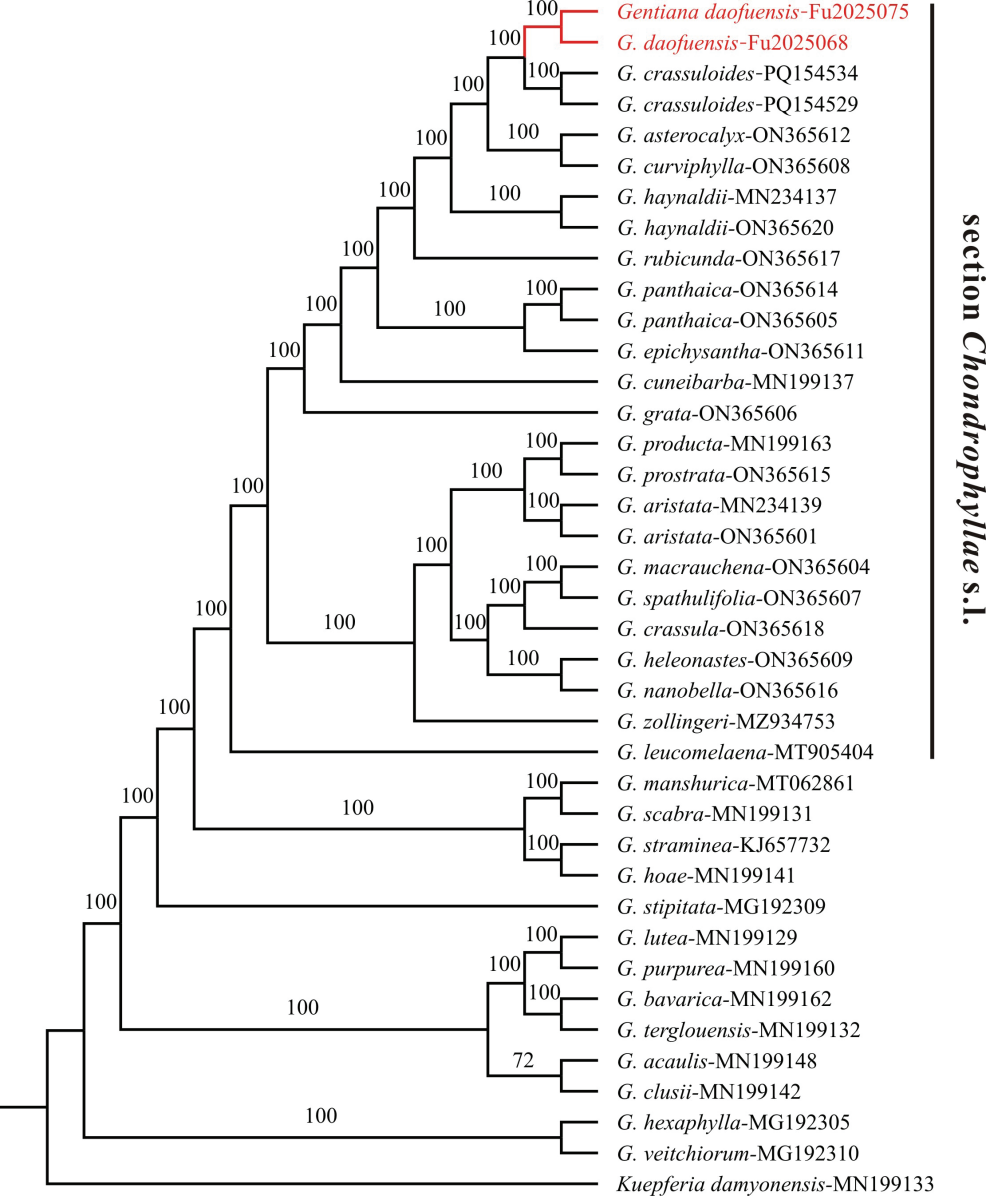
Phylogenetic tree of *Gentiana* section *Chondrophyllae* s.l. based on chloroplast genomes. Bootstrap support values obtained from maximum likelihood analyses are presented above the branches.

The two newly sequenced chloroplast genomes of *G. daofuensis* range from 128,253 to 128,263 bp in length, with a GC content of 37.4%. The lengths of the Large Single Copy (LSC), Small Single Copy (SSC), and IR are 73,073–73,082 bp, 10,421–10,480 bp, and 22,370–22,381 bp, respectively. The lengths of the total chloroplast genome, LSC, SSC, and IR of *G. daofuensis* fell within the average range of section *Chondrophyllae* but were much shorter than those of other sections in *Gentiana* (Sun et al. 2018; Fu et al. 2022). Phylogenetic analysis included these sequences alongside 36 previously published *Gentiana* chloroplast genomes, 23 of which belong to *G.* section *Chondrophyllae* s.l. The resulting chloroplast genome alignment produced a well-supported phylogenetic tree, with nearly all nodes receiving full support (100% bootstrap support; Fig. 3). All sampled species of *G.* section *Chondrophyllae* s.l. formed a monophyletic clade. The two *G. daofuensis* samples clustered together with *G. crassuloides*, and this group was sister to *G. curviphylla* and *G. asterocalyx* Diels (Fig. 3).

#### Discussion.

In his book “Gentians of the World,” Dr. Yong-Ming Yuan described a new *Gentiana* species named *G. daofuensis* (Yuan 2024); however, that work did not provide direct morphological or genetic evidence, nor did it designate type material. Based on field observations and specimen comparisons of *G. daofuensis* and its close relatives, our results clearly demonstrate that *G. daofuensis* differs from *G. crassuloides* in corolla color, calyx length, and habitat preference and from *G. curviphylla* in leaf shape and corolla color (Table 1; Fig. 2). Consequently, these three species can be reliably distinguished despite their sympatric occurrence in western Sichuan Province. Phylogenetic analysis incorporating multiple individuals of the target species placed *G. daofuensis* in a distinct, fully supported position in the molecular phylogenetic tree, confirming its status as a separate species in section *Chondrophyllae* s.l. Based on its morphological traits, *G. daofuensis* belongs to ser. *Orbiculatae*, which is characterized by ovate calyx lobes with a narrowed base.

#### Additional specimens examined (paratypes).

*Gentiana daofuensis*. China • Sichuan: Daofu County, near Taining Village, alt. 3650 m, 101.54°N, 30.54°E, 23 Jul 2025, flowering and with few fruits, P.C. Fu & S.S. Sun *Fu2025068* (HNWP392647!; herbarium of Luoyang Normal University); • Daofu County, near Geka Village, alt. 3810 m, 101.31°N, 30.78°E, 12 Sept 2023, in flower and fruit, Y.M. Yuan *Fu2023089* (herbarium of Luoyang Normal University).

## Supplementary Material

XML Treatment for Gentiana daofuensis

## Additional information

### Use of AI

For this submission, AI tools were used solely for language polishing and refinement. Specifically, AI-assisted grammar checking, sentence structure adjustment, and word choice optimization were applied to enhance the overall clarity, fluency, and academic tone of the manuscript. This assistance was used throughout the entire paper to ensure the English text was precise and readable, while all scientific content, data analysis, interpretations, and conclusions remain entirely our own original work.

### Author ORCIDs

Peng-Cheng Fu https://orcid.org/0000-0002-6201-0808

### Data availability

The plastome dataset supporting the conclusions of this article is accessible at: https://github.com/fupengc/Plastome-data-in-phylogeny-of-~~~PROTECTED_TN_76~~~-daofuensis.
